# The effect of dexamethasone as an adjuvant in spinal anesthesia for femur upper extremity surgery: a prospective randomized trial

**DOI:** 10.11604/pamj.2022.43.29.36117

**Published:** 2022-09-19

**Authors:** Ahlem Bousabbeh, Salma Ketata, Nizar Sahnoun, Mariem Keskes, Omar Ketata, Wiem Ben Amar, Imen Zouche, Abdelhamid Karoui

**Affiliations:** 1 Department of Anesthesiology and Intensive Care Unit, Habib Bourguiba University Hospital, 3000 Sfax, Tunisia,; 2 Department of Orthopedic Surgery, Habib Bourguiba University Hospital, 3000 Sfax, Tunisia,; 3 Departement of Legal Medicine, Habib Bourguiba, Sfax University Hospital, 3000 Sfax, Tunisia

**Keywords:** Spinal anesthesia, dexamethasone, sensory bloc, pain assessment

## Abstract

**Introduction:**

the aim of our study was to evaluate the efficacy of dexamethasone added to bupivacaine and sufentanil in spinal anesthesia to improve postoperative analgesia after femur upper extremity surgery.

**Methods:**

we conducted a prospective controlled, randomized double-blinded clinical trial including patients proposed for surgery of the upper extremity of the femur under spinal anesthesia. The patients were randomly allocated to receive intrathecally 10 mg hyperbaric bupivacaine 0.5% with 5µg sufentanil and 2 ml normal saline (control group) or 10 mg hyperbaric bupivacaine 0.5% with 5 µg sufentanil and 8 mg dexamethasone (Dexa group). The patients were evaluated for onset time and duration of sensory block, duration of pain-free period, overage consumption of morphine in the 6 first postoperative hours, hemodynamic parameters, nausea, and vomiting, or other complications.

**Results:**

fifty-eight patients were analyzed. There were no signification differences in demographic data and onset time of the sensory block between the two groups. Sensory block duration was 121.55 ± 16.42 minutes in the control group and 183.62 ± 33.93 minutes in the Dexa group which was significantly higher in the Dexa group (P<0.001). The pain-free period was longer in the Dexa group than in the control group (P<0.001). There was a reduction in morphine consumption during the first 6 postoperative hours in the Dexa group against the control group (p=0.02). The frequency of complications was not different between the two groups.

**Conclusion:**

the addition of intrathecal dexamethasone in spinal anesthesia improved the postoperative analgesia after femur upper extremity surgery.

## Introduction

Fractures of the upper extremity of the femur affect frequently elderly patients and are responsible for significant morbidity and mortality [1]. Optimal management of these lesions is decisive for the quality of functional results. The anaesthetic technique of choice is spinal anaesthesia (SA) [2]. However, its duration, as well as postoperative analgesia, remains relatively limited. The addition of intrathecal (IT) adjuvants to local anaesthetics to improve and prolong per and postoperative analgesia is increasingly used [3]. Dexamethasone has shown its efficacy and harmlessness as an adjuvant to local anaesthetics [4,5] but has not benefited from sufficient experimentation in spinal anaesthesia. The aim of the study was to evaluate the efficacy of the addition of dexamethasone to bupivacaine and sufentanil for spinal anaesthesia to improve postoperative analgesia in patients undergoing surgery on the upper extremity of the femur.

## Methods

We conducted a prospective controlled double-blinded randomized clinical trial in the anaesthesia-intensive care unit involving patients proposed for surgery of the upper extremity of the femur under spinal anaesthesia (SA).

### Study population

**Inclusion criteria were:** patients older than 40 years old (since the fracture of the upper extremity of the femur affects the elderly patients) with an American Society of Anesthesiologists (ASA) score of I or II, candidates for surgery of the upper extremity of the femur proposed for DHS (Dynamic Hip Scrue) under spinal anaesthesia (SA).

Non-inclusion criteria were: absolute contraindications to spinal anaesthesia, the patient's refusal to be included in the study, known allergy to dexamethasone or local anaesthetics, and misunderstanding of the visual analogue scale.

**The exclusion criteria were:** technical difficulties in performing SA, partial or total failure of the SA requiring conversion to General Anesthesia (GA), a severe complication of anaesthesia, or surgery requiring the conversion to general anaesthesia.

**Sample size:** since there was no clinical trial investigating the effect of intrathecal dexamethasone on reducing postoperative pain after fracture of the upper extremity of the femur, the sample size was calculated from a previous pre-survey of 20 patients, that we did, showing that the mean duration of sensory block in the dexa group was 185.50 ± 29.64, while mean sensory block in the control group was 119.43 ± 15.20 setting alpha at 5% and power of study 90%. Calculation according to these values produced a minimal sample size of 16 patients in each group. We then designated 30 patients per group to have sufficient numbers after possible exclusions.

### Randomization and allocation

Patients were randomized automatically according to a sequence generated by the website: www.sealedenvelope.com into two groups: Dexa group (patients who received intrathecal dexamethasone added to bupivacaine and sufentanil) and control group (patients who received intrathecal normal saline added to bupivacaine and sufentanil. Interventions).

In the operating room, patients were monitored by an electro-cardioscope, pulse oximetry, and non-invasive blood pressure monitoring. An 18-Gauge vein needle was placed and patients received 10 cc/kg of 0.9% of isotonic saline. Spinal anaesthesia was performed in the sitting position at L4-L5 or L5-S1 intervertebral space through a midline approach using a 25-gauge Quincke spinal needle. Patients of the control group received 10 mg (2 ml) of 0.5% hyperbaric bupivacaine with 5 µg of sufentanil (1 ml) diluted in preservative-free normal saline (2 ml) and patients of the Dexa group received 10mg (2 ml) of 0.5% hyperbaric bupivacaine with 5 µg of sufentanil (1 ml) and 8 mg preservative-free dexamethasone (2 ml). Overall 5 ml volume was administrated intrathecally in each group. The dexamethasone used in this study was the UNIDEX©. To facilitate the double-blinding method, the intrathecal solution was prepared by the anesthesiologist who was responsible for the randomization and injected slowly by a second anesthesiologist who was blinded to the nature of the solution. After the spinal anaesthesia, patients who also didn't know the nature of the solution were kept in the supine position, and oxygen of 3 L/ min was given through a face mask. The sensory block defined by the patient´s inability to feel pain and not differentiate between hot and cold was evaluated by the thermal method using a compress soaked with ether every two minutes until the installation of a metameric level at D6 for the authorization of the surgery. If after 20 minutes the level is insufficient, conversion to general anaesthesia was performed and the patient was excluded from the study.

The onset time of the sensory block was defined from the time of injection of drugs into the intrathecal space to the peak of sensory block (highest dermatome level) and the duration of sensory block was defined from a peak of sensory block up to 4 sensory level regressions or when the patients feel pain in the field of surgery. Intraoperative hemodynamic parameters were collected every 20 minutes until the end of the procedure. After the surgery, the patient was transferred to the Post Interventional Monitoring Room (PIMR) for 6 hours where standard monitoring was established. Hemodynamic parameters, pain assessment, and side effects were collected. The sensory block was re-evaluated until total regression. For the pain assessment, we used the visual analogue pain scale (VAS) previously explained to the patient. Postoperative analgesia was provided by: 1 g of paracetamol and 2 mg Morphine titration every 5 minutes if the VAS value was greater than 3. After that, patients were transferred to their referral department. They were examined every day before leaving the hospital and 1 month later and asked about any postoperative complications.

**Data collection:** pre- and intraoperative data were collected including patients´ demographics, ASA scores, and duration of surgery. We collected anaesthetic and analgesic parameters such as the onset time and the duration of the sensory block and pain-free period, VAS at the 2^nd^, 6^th^, 12^th^ and 24^th^ postoperative hours, the overage dose of morphine consumption, and the hemodynamic parameters (heart rate, systolic, diastolic arterial pressure) every 20 minutes for the first two postoperative hours then at the 2^nd^, 6^th^, 12^th^ and 24^th^ postoperative hours. The collection of post-operative data was done by a third anesthesiologist who didn´t know the randomized groups.

**Variables:** our primary outcome was the duration of the sensory block defined by the duration of inability to feel pain and not differentiate between hot and cold. Our secondary outcomes were the onset time of the sensory block, the pain-free period after surgery, the overage dose of morphine consumption for the first 6 postoperative hours, and the side effects related to SA and intrathecal dexamethasone (nausea, vomiting, hypotension, bradycardia, shivering, headache or dyspnea).

**Statistical analysis:** SPSS version 25.0 software was used for data analysis. We checked the normality of the distribution by the Shapiro-Wilk test for the quantitative variables. Continuous variables and data with a normal distribution are expressed as means (SD) and as medians with the semi-interquartile ranges (SIR) otherwise. Qualitative variables were expressed as frequency distributions. Univariate comparisons between the two groups of patients were performed using t- student test, Mann-Whitney test, and Pearson´s Chi-square test. Statistical significance was defined as p < 0.05.

**Ethical considerations:** this study was conducted after approval of the southern protection committee of people (C.P.P.SUD) under the aegis of the Health Ministry of the Tunisian republic reference CPP SUD N°0215/2020for the nature of the product and the conduct of the study. This study was conducted after the written informed consent of patients.

**Finding statements:** we had no financial support for this work.

## Results

**Figure 1:**
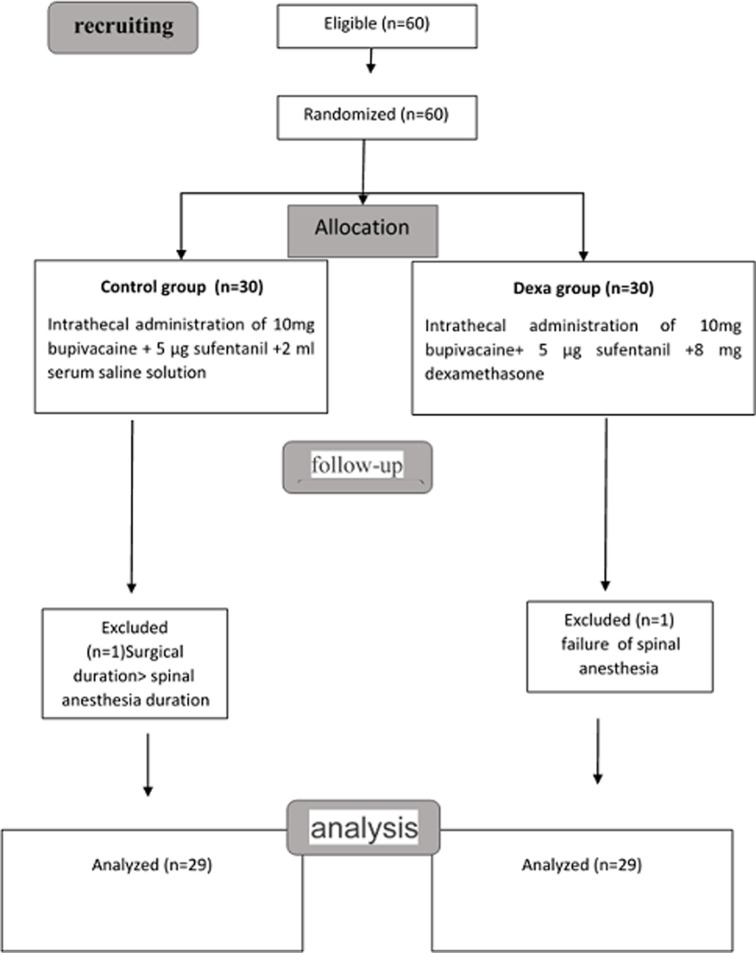
flow chart

Sixty patients were included in our study and were divided into two equivalent groups. Two patients were excluded for a failure of spinal anaesthesia and a surgical duration longer than the spinal anaesthesia duration. The number of patients analyzed was 58 with 29 patients in each group (Figure 1).

**General characteristics:** our sample was characterized by a mean age = 71.21 ± 13.8 years, a sex ratio (male/female) =1.15 and a mean BMI = 28.775 ± 7.401 kg/m^2^. There was no statistical difference between the Dexa and control groups in terms of demographic data, comorbidities, and duration of surgery (Table 1).

**Table 1:** comparison of demographic data between the 2 groups

	Control group (N=29)	Dexa group (N=29)	P-value
**Age (years) ± SD**	72.45±12.246	69.97 ± 15.47	0.5*
**weight(kg) ± SD**	76.83 ± 10.80	72.66 ± 12.28	0.17*
**High (cm) ± SD**	1.64 ± 0.04	1.611 ± 0.13	0.25*
**BMI(kg/cm^2^) ± SD**	28.48 ± 3,78	29.07 ± 11,02	0,78*
**Sexe(M/F)**	15/14	16/13	0,79ƚ
**ASA score (I/II)**	8/21	9/20	0,773ƚ
**Surgery duration (mn) ± SD**	80,17 ± 15,725	75,17 ± 24,838	0,364*

SD: standard deviation, mn: minutes, cm: centimeter, Kg: kilogram *: t-student test, ƚ: Pearson´s Chi-square test

**Postoperative analgesia:** there was no significant difference in the mean of the onset time of the sensory block between the 2 groups. It was 5.62 ± 1.49 minutes for the Dexa group and 5.14 ± 1.02 minutes for the control group (P= 0.15). The duration of the sensory block was 121.55 ± 16.42 minutes in the control group and 183.62 ± 33.93 minutes in the Dexa group with a P-value less than 0.001. The pain-free period in the Dexa group was 273.1 ± 33.97 minutes was longer than that in the control group151.72 ± 27.78 with a P-value less than 0.001 (Table 2). The comparison of VAS (Visual Analogue Pain Scale) during the first 24 postoperative hours between the two groups showed a significant difference at all measurement points in favour of the Dexa group compared to the control group (Table 3). In our study, we showed a reduction in the morphine consumption during the first 6 postoperative hours in the Dexa group of 0.21 mg [0-4] mg against the control group of 1.8 mg [0-6] with p=0.02 (Table 2)

**Table 2:** comparison of onset time and duration of sensory block, pain-free period and morphine consumption between the 2 groups

Parameters	Control group	Dexa group	P-value
**Onset time (mn) ± SD**	5.14 ± 1.02	5.62 ± 1.49	0.15 *
**Duration of sensory block (mn) ± SD**	121.55 ± 16.42	183.62 ± 33.93	<0,001*
**Pain-free period (mn) ± SD**	151.72 ± 27.78	273.1 ± 33.97	<0,001*
**average dose of morphine (mg) [1Q-3Q]**	1,8 [0-6]	0,21 [0-4]	0,002¥

mn: minutes, mg: milligram, SD: standard deviation, 1Q: lower quartile; 3Q: upper quartile *: t-student test, ¥: Mann-Whitney test

**Table 3:** comparison of VAS between the 2 groups

Parameters	Control group	Dexa group	P value
**VAS (H2) [1Q-3Q]**	3,28 [2-4]	0,25 [0-2]	<0,001¥
**VAS (H6) [1Q-3Q]**	4,05 [2-6]	2,04 [1-3]	<0,001¥
**VAS (H12) [1Q-3Q]**	2,88 [2-4]	2,24 [1-4]	<0,001¥
**VAS (H24) [1Q-3Q]**	2,48 [2-4]	1,78 [1-3]	<0,001¥

1Q: lower quartile; 3Q: upper quartile, ¥: Mann-Whitney test

**Side effects:** there was no significant difference in heart frequency, systolic blood pressure, and diastolic blood pressure intra and the post-operative period between the two groups. No postoperative neurological deficit or allergic reactions were observed in any patients. There was no significant difference between the 2 groups in terms of other postoperative complications such as itching, nausea, and vomiting (Table 4).

**Table 4:** comparison of side effects between the 2 groups

Side effects	Control group	Dexa group	P value
**post-operative nausea and vomiting (P/A)**	3/26	1/28	0,118ƚ
**itching (P/A)**	4/25	5/24	0,5ƚ

P/A: present/absent, ƚ : Pearson´s Chi-square test

## Discussion

Our study is one of the few studies in the literature to have used Dexamethasone as an adjuvant to SA. We conducted a prospective double-blinded randomized clinical trial including patients proposed for upper femur surgery under spinal anaesthesia. Fifty-eight patients were analyzed and randomized into two groups of 29. This study showed that dexamethasone significantly prolonged the analgesic effect of bupivacaine and sufentanil in spinal anaesthesia, without any effects on the onset time of the sensory block, or altering hemodynamic stability and without the occurrence of side effects. Several experiments demonstrated the analgesic effects of steroids in neuraxial and peripheral blocks.

Bani-Hashem *et al*. [6], used dexamethasone in patients who had orthopaedic surgery under spinal anaesthesia. This study showed that the addition of intrathecal dexamethasone to bupivacaine significantly improved the duration of the sensory block and increased the time of the first analgesic intake without affecting the onset time of the sensory block. Šakic *et al*. [7], showed in a prospective randomized clinical trial involving 60 patients with ASA2 and ASA3 status, programmed for femur fracture, that the addition of dexamethasone to the anaesthetic improved the quality of the analgesia. Fayyaz *et al*. [8] showed in a prospective study including 60 patients proposed for the cesarean section that the addition of intrathecal dexamethasone to bupivacaine hyperbaric significantly increased the duration of analgesia compared to bupivacaine hyperbaric.

Kotani *et al*. [9] administered methylprednisolone with bupivacaine intrathecally in patients with post-zosterian neuralgia. They concluded that this combination induced excellent and lasting analgesia. Several other studies have shown an extension of the duration of the sensory block in case of the addition of Dexamethasone to different local anaesthetics: a recent meta-analysis done in 2017 by Kyle Robert Kirkham *et al*. [10] including 33 clinical trials and 2138 patients, showed that the duration of the sensory and motor block of the brachial plexus was statistically significantly extended by Dexamethasone with a maximum dose of 4mg. Another meta-analysis from 2015, conducted by Albrecht *et al*. [11], covering 29 clinical trials and 1,659 patients, showed the same results. Several other studies [12-17] have demonstrated the analgesic prolongation of peripheral blocks of the upper limb following the addition of Dexamethasone.

In our study, we found that intrathecal dexamethasone does not increase the hypotension induced by local anaesthetics. This is consistent with the literature. Jabbari *et al*. [18], and El shourbagy *et al*. [19] showed in a study combining dexamethasone and bupivacaine in IT versus bupivacaine alone that there was no significant difference between the two groups in terms of hypotension episodes. In our study, no neurological complications were noted in both groups. Several studies in vitro or in vivo have evaluated the incidence of neurological complications related to the use of peri-nerve dexamethasone. No nerve damage was found in the various studies [20-22]. Moreover, a wide review of recent literature has not found any cases of neurotoxicity reported after perineural injection of dexamethasone as an adjuvant to the peripheral nerve block in 29 controlled trials involving 1,695 participants [11].

**Limitation:** it was impossible to operate on patients with the same surgeon and there is inter-individual variability in the pain perception. Future studies comparing the same dose of dexamethasone administered in IT and systemically will be necessary to recommend the use of dexamethasone in IT in daily clinical practice.

## Conclusion

The addition of intrathecal dexamethasone to bupivacaine and sufentanil significantly improved the duration of sensory block in spinal anesthesia, increased the pain-free period, and decreased opioid requirements in postoperative management without side effects.

### What is known about this topic

- Dexamethasone is a synthetic glucocorticoid;
- Dexamethasone, given intravenously, has analgesic and anti-inflammatory effects;
- Dexamethasone administered perineurally as an adjuvant to locoregional anesthesia significantly lengthens the duration of the sensory block.

### What this study adds

- The addition of intrathecal dexamethasone to bupivacaine and sufentanil in spinal anesthesia: improved the duration of sensory block; increased the pain-free period; decreased opioid requirements in postoperative management; does not increase side effects related to spinal anesthesia.
